# Kaposi sarcoma incidence in the Swiss HIV Cohort Study before and after highly active antiretroviral therapy

**DOI:** 10.1038/sj.bjc.6604520

**Published:** 2008-07-29

**Authors:** S Franceschi, L Dal Maso, M Rickenbach, J Polesel, B Hirschel, M Cavassini, A Bordoni, L Elzi, S Ess, G Jundt, N Mueller, G M Clifford, and the Swiss HIV Cohort Study

**Affiliations:** 1 International Agency for Research on Cancer, 150 cours Albert Thomas, 69372 Lyon cedex 08, France; 2 Epidemiology and Biostatistics Unit, Aviano Cancer Center, Via Franco Gallini 2, 33081 Aviano, Italy; 3 Coordination and Data Center, Swiss HIV Cohort Study, Mont-Paisible 16, CHUV, 1011 Lausanne, Switzerland; 4 Division of Infectious Diseases, Department of Internal Medicine, University Hospital of Geneva, Rue Michel-du-Crest 24, CH-1211 Geneva 14, Switzerland; 5 Division of Infectious Diseases, Department of Medicine 2, CHUV Lausanne, Lausanne 1011, Switzerland; 6 Cancer Registry of the Canton of Ticino, Via in Selva 24, CH-6600 Locarno, Switzerland; 7 Division of Infectious Diseases and Hospital Epidemiology, University Hospital Basel, Petersgraben 4, CH-4031 Basel, Switzerland; 8 Cancer Registry of St Gallen and Appenzell, PO Box 2, CH-9007 St Gallen, Switzerland; 9 Cancer Registry of Basel, Schönbeinstrasse 40, CH-4003 Basel, Switzerland; 10 Division of Infectious Diseases and Hospital Epidemiology, Department of Medicine, University Hospital Zurich, Rämistrasse 100, CH-8091 Zurich, Switzerland

**Keywords:** HIV, AIDS, Kaposi sarcoma, antiretroviral drugs, Swiss HIV cohort study

## Abstract

Between 1984 and 2006, 12 959 people with HIV/AIDS (PWHA) in the Swiss HIV Cohort Study contributed a total of 73 412 person-years (py) of follow-up, 35 551 of which derived from PWHA treated with highly active antiretroviral therapy (HAART). Five hundred and ninety-seven incident Kaposi sarcoma (KS) cases were identified of whom 52 were among HAART users. Cox regression was used to estimate hazard ratios (HR) and corresponding 95% confidence intervals (CI). Kaposi sarcoma incidence fell abruptly in 1996–1998 to reach a plateau at 1.4 per 1000 py afterwards. Men having sex with men and birth in Africa or the Middle East were associated with KS in both non-users and users of HAART but the risk pattern by CD4 cell count differed. Only very low CD4 cell count (<50 cells *μ*l^−1^) at enrolment or at HAART initiation were significantly associated with KS among HAART users. The HR for KS declined steeply in the first months after HAART initiation and continued to be low 7–10 years afterwards (HR, 0.06; 95% CI, 0.02–0.17). Thirty-three out of 52 (63.5%) KS cases among HAART users arose among PWHA who had stopped treatment or used HAART for less than 6 months.

At the beginning of the HIV epidemic, Kaposi sarcoma (KS) was one of the most common manifestations of AIDS (Dal Maso *et al*, 1995; Biggar *et al*, 1996), present during the mid-1980s in 25% of individuals at the time of AIDS diagnosis in the United States, but decreased steadily through the late 1980s and mid-1990s, down to 2% after the advent and widespread use of highly active antiretroviral therapy (HAART) in 1996 (Engels *et al*, 2006).

A similar temporal pattern was observed for KS in Australia (Grulich *et al*, 2001) and in European countries (Dal Maso *et al*, 1995; Rezza *et al*, 2000; Franceschi *et al*, 2003; Mocroft *et al*, 2004; Clifford *et al*, 2005), but detailed data on the long-term trends of KS incidence in Europe are limited. We therefore took advantage of the more than 20 years of follow-up data available from the Swiss HIV Cohort Study (SHCS) to assess changes in the incidence of and risk factors for KS before and after HAART use.

## Materials and methods

The SHCS is an ongoing study that has been enrolling people with HIV/AIDS (PWHA) over 16 years of age since 1988, with some retrospective enrolment going back to 1984, from seven large hospitals in Swiss cities (Basel, Bern, Geneva, Lausanne, Lugano, St Gallen, and Zurich) (www.shcs.ch). Follow-up visits take place every 6 months and all AIDS-defining events, including KS diagnosis and death, are recorded. The present study included PWHA enrolled up to 30 September 2005, and information recorded in the SHCS database up to 31 March 2006. People with HIV/AIDS were excluded from the present study if they (1) did not have information on date of birth, gender, or HIV transmission category (number (*n*)=54), (2) were diagnosed with KS at enrolment or earlier (*n*=368), or (3) had no follow-up visits (*n*=131).

A total of 597 KS cases were included in our present study: 545 were identified from the SHCS database, and 52 through record linkage with eight Swiss Cantonal Cancer Registries (Clifford *et al*, 2005). Six of these cancer registries (Basel, Geneva, Ticino, St Gallen and Appenzell, Vaud, and Zurich) overlap directly with six of the seven cantons covered by SHCS hospitals (all except Bern). The Neuchâtel and Valais Cancer Registries do not directly overlap with SHCS hospitals, but some residents of these cantons are followed in a neighbouring SHCS hospital. Places of birth were classified as Europe (Switzerland and the rest of Europe, 87.1% of PWHA) and Africa or the Middle East (8.1%). The few SHCS participants born outside Europe, but in countries where KS is not endemic (e.g., the Americas and Asia; Hengge *et al*, 2002), were included in the Europe category. Conversely, the few PWHA born in the Caribbean were included in the Africa/Middle East category. Histological confirmation was mentioned in the majority of KS cases, but presentation site (i.e., skin only *vs* other) was available only for 382 (64%) KS cases.

Highly active antiretroviral therapy was defined as a combination of at least three drugs, including a protease inhibitor or a non-nucleoside transcriptase inhibitor, or three nucleosides including abacavir. Individuals who had used HAART for more than 1 month were classified as users. Treatment interruption was defined as in a previous report from the SHCS (Taffé *et al*, 2002), as absence of any antiretroviral drug in PWHA who were previously receiving HAART. Taffé *et al* (2002) evaluated the impact of interruptions of less than 3 months on the progression of HIV infection, whereas we focused on the impact of interruptions of 3 months or more on KS incidence. CD4 cell counts at enrolment in the SHCS and, among HAART users, at, or within 6 months before HAART initiation were retrieved.

For each participant, person-years (py) at risk were calculated between enrolment and KS diagnosis, death, or last follow-up visit, whichever occurred first. Incidence rates per 1000 py were standardised for gender and age based on the enrolled population in the overall study period, using the direct method (Breslow and Day, 1987). Ninety-five percent confidence intervals (CI) of incidence were computed according to the Poisson distribution (Breslow and Day, 1987). The effect of various risk factors on KS onset was assessed using hazard ratios (HR) and corresponding 95% CI, estimated by means of the Cox proportional hazard model (Cox, 1972), and adjusted for SHCS centre, gender, age (in 5-year groups), HIV transmission category (MSM and non-MSM) and, when mentioned, CD4 cell count at enrolment or at HAART initiation (<50, 50–99, 100–199, 200–349, ⩾350 cells *μ*l^−1^, and unknown). Calendar period, HAART use, and months after HAART initiation and after treatment interruption were introduced as time-dependent variables.

This study was approved by the local ethics committees of the collaborating SHCS clinics and of the International Agency for Research on Cancer.

## Results

The present study included 12 638 PWHA who were KS-free at enrolment and among whom 597 incident KS cases were identified (8.2 per 1000 py; 95% CI, 7.6–8.9). Fifteen per cent of PWHA had AIDS at enrolment and an additional 3119 (24.7%) developed it during follow-up. Among the latter, KS was the AIDS-defining illness in 268, whereas 329 KS cases developed in PWHA who had already manifested another AIDS-defining illness.

**Figure 1:**
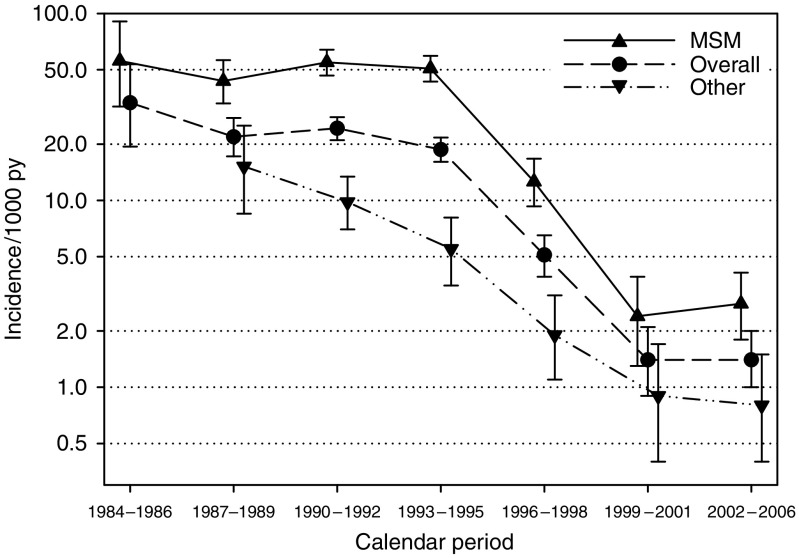
Incidence rates of KS by calendar period, overall and according to HIV transmission category. Rates were standardised (direct method) on age and gender, based on Swiss HIV Cohort Study participants. Vertical bars represent 95% CI. MSM: men having sex with men.

Figure 1 shows KS temporal trends: overall KS incidence was 33.3 per 1000 py in 1984–1986 and did not change significantly in the subsequent periods until 1996–1998, when it fell to 5.1 (95% CI, 3.9–6.5) per 1000 py. Kaposi sarcoma incidence further decreased to 1.4 per 1000 py in 1999–2001 and remained constant thereafter. Temporal trends in KS incidence were chiefly driven by men having sex with men (MSM), but they were consistent among other HIV transmission categories (Figure 1).

A large proportion (48.4%) of available py derived from HAART users and Table 1 shows the incidence and HR of KS by various characteristics separately among non-users and users of HAART. Incidence of KS decreased from 15.0 per 1000 py in non-users to 1.3 per 1000 py in users (HR, 0.11; 95% CI, 0.08–0.14). Among non-users of HAART, intravenous drug users (HR, 0.09; 95% CI, 0.06–0.13), and heterosexuals and other HIV transmission categories (HR, 0.27; 95% CI, 0.20–0.36) showed a lower KS incidence than MSM. The HR for KS was increased among PWHA older than 35 years (HR, 1.53; 95% CI, 1.29–1.82) and those born in Africa/Middle East (HR, 1.84; 95% CI, 1.10–3.06). Kaposi sarcoma risks among non-users of HAART steeply increased with decreasing CD4 cell count (HR for <50 *vs* ⩾350 cells *μ*l^−1^, 12.85; 95% CI, 9.59–17.23). These associations were also present, but were weaker, among HAART users with the exception of the association with place of birth that became stronger (HR for Africa/Middle East *vs* Europe among HAART users, 6.49; 95% CI, 2.79–15.11). In contrast with non-users, no change in the HR for KS was seen among HAART users with CD4 cell counts in the range of 50–⩾350 cells *μ*l^−1^ and the only significant risk increase was found for CD4 cell count less than 50 cells *μ*l^−1^ at enrolment (HR, 3.26; 95% CI, 1.53–6.91). On account of the rarity of KS among HAART users, HRs showed, however, broad CIs (Table 1).

Among HAART users, CD4 cell count at treatment initiation below 50 cells *μ*l^−1^ (HR *vs* ⩾350 cells *μ*l^−1^, 5.36; 95% CI, 2.08–13.80) (data not shown) was even more strongly associated with KS risk than CD4 cell count at enrolment. Furthermore, KS risk was greatly increased among PWHA who had stopped using any antiretroviral drugs for at least 3 months (HR, 8.14; 95% CI, 4.01–16.54) (Table 1). Additional adjustment for CD4 cell count at HAART initiation did not modify the HR for treatment interruption (HR, 9.45; 95% CI, 4.64–19.25, data not shown).

Skin was reported as the presentation site in the majority of KS cases (74.3%). Temporal trends and associations with HAART use and CD4 cell count (overall and in separate strata by HAART use) did not differ by KS presentation site (data not shown).

**Figure 2:**
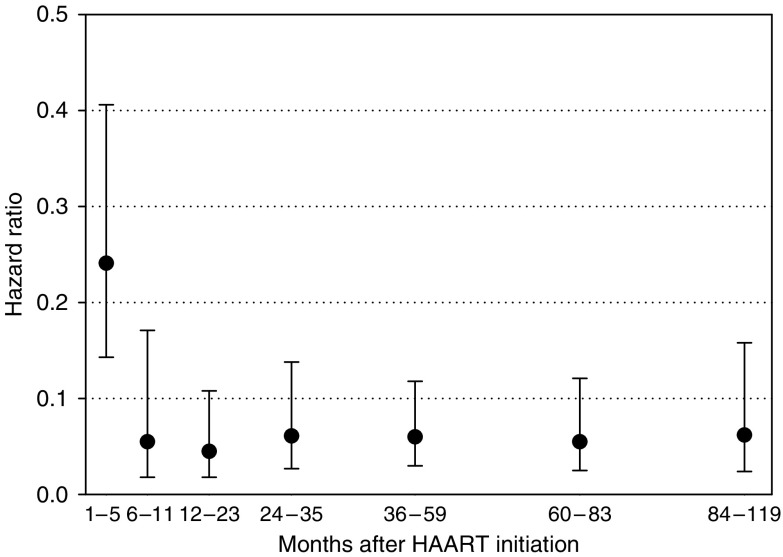
Hazard ratio of Kaposi sarcoma in patients receiving highly active antiretroviral therapy (HAART) following treatment initiation. Adjusted for centre, age, gender, HIV transmission category (men having sex with men, other), and CD4 cell count at enrolment. Vertical bars represent 95% confidence intervals. Reference category was defined as non-users of HAART.

Figure 2 shows the HR for KS in different periods after HAART initiation compared with non-users. The HR of KS was already reduced by 76% in the first 5 months of use and declined to 0.06 (95% CI, 0.02–0.17) in the subsequent 6 months of use. The reduction in KS risk persisted unchanged up to 84–119 months after HAART initiation (HR, 0.06; 95% CI, 0.02–0.16) (Figure 2).

Finally, the 52 HAART users who developed KS were individually reviewed and classified into the following groups: (1) no antiretroviral drug in the 3 months before KS diagnosis; (2) recent initiation of HAART (<6 months before KS diagnosis); (3) severe immunodeficiency (CD4 cell count <100 cells *μ*l^−1^ at KS onset while on HAART for ⩾6 months); and (4) none of the above (data not shown). Eighteen (34.6%) KS cases had not been taking any antiretroviral drug for 3 months or more, and in 10 instances for 12 months or longer. Recent initiation of HAART was identified in 15 KS cases, among whom five of the nine KS were from Africa/Middle East. Severe immunodeficiency was identified among 10 KS cases. Nine KS cases, all from the MSM transmission category, could not be assigned to any of the three categories above. Five of them (aged 35, 49, 52, 56, and 63 years) had a CD4 cell count ⩾400 cells *μ*l^−1^ (i.e., 405, 440, 557, 596, 782) at KS diagnosis.

## Discussion

Our study shows the dramatic decline of KS incidence in the SHCS following the advent of HAART. This expands an earlier report (Ledergerber *et al*, 1999) from this cohort showing that by 1998 the KS decline was at least as large as that seen for opportunistic infections. Similar reductions in the incidence of KS among PWHA were seen in many other studies (International Collaboration on HIV and Cancer, 2000) although the decrease started earlier (i.e., even before the introduction of HAART) in the United States (Biggar *et al*, 1996; Engels *et al*, 2006) and Australia (Grulich *et al*, 2001) than in Europe (Dal Maso *et al*, 1995). The prevalence of KS herpesvirus, the cause of KS (IARC, 1997), may have been especially high in the first wave of HIV infection in MSM in the United States and Australia (Osmond *et al*, 2002).

Highly active antiretroviral therapy became rapidly available to SHCS participants and, by 1997, 80% of them were using three antiretroviral drugs or more (www.shcs.ch). The proportion of SHCS participants with CD4 cell counts <350 cells *μ*l^−1^ who had never been treated was small (<3%) in 2006 in all HIV transmission categories. Despite the widespread use of HAART and the introduction after 1996 of successively more potent antiretroviral drugs, KS incidence in the SHCS seems to have reached a plateau after 2001, as reported among AIDS patients in the United States (Engels *et al*, 2006).

In addition to making KS a relatively rare event, HAART use has also diminished the variation in KS risk by host characteristics, including gender, age group, and to some extent, HIV transmission category and CD4 cell count at enrolment as compared with that found among non-users. Only a count <50 cells *μ*l^−1^ at enrolment or HAART initiation was associated with an increased HR for KS. Reduced importance of CD4 cell count at enrolment in HAART users *vs* non-users was also seen in the SHCS for non-Hodgkin's lymphoma, but the impact of HAART on non-Hodgkin's lymphoma was weaker (HR, 0.26; 95% CI, 0.20–0.33) than on KS, and, hence, non-Hodgkin's lymphoma incidence (1.9; 95% CI, 1.6–2.6 per 1000 py) became higher than KS incidence among HAART users (Polesel *et al*, 2008).

Kaposi sarcoma risk was already reduced by over 90% after 1 year of HAART and it did not show any sign of increasing again for at least 10 years. The decline of non-Hodgkin's lymphoma risk after HAART initiation was more gradual than for KS, but equally prolonged (Polesel *et al*, 2008).

Approximately one-third of HAART users in the SHCS had one or more interruptions of antiretroviral treatment (Taffé *et al*, 2002) due in most cases either to intolerance to drugs, or social factors (i.e., being an intravenous drug user, poor education, etc.), and not to treatment failure. In our study, the absence of any antiretroviral treatment for 3 months or more was associated with an eight-fold increased KS risk, thus confirming the danger of treatment interruption already reported with respect to progression to AIDS or death (Holkmann *et al*, 2007). Significantly higher KS incidence among PWHA who were assigned to the CD4 cell-guided intermittent antiretroviral treatment arm than those assigned to the continuous treatment arm was also shown in a randomised clinical trial (Silverberg *et al*, 2007).

Of the 52 KS among HAART users, 23 arose among people who had either stopped using HAART at or had initiated treatment less than 6 months before KS diagnosis. Recent initiation of HAART in the SHCS seemed especially important among KS cases born in Africa/Middle East, suggesting possible delays in the diagnosis or treatment of HIV infection. Ten KS cases arose in PWHA whose CD4 cell count was very low despite concurrent HAART use whereas 5 MSM developed KS despite being on HAART and having CD4 cell counts at which AIDS-related KS is seldom seen (Biggar *et al*, 2007). The occurrence of KS cases in PWHA with high CD4 cell counts and undetectable viral loads has already been reported in the United States after 1996 (Maurer *et al*, 2007; Krown *et al*, 2008). It is possible that, with the ageing of PWHA, those who are co-infected with KS herpesvirus may develop KS despite good control of HIV infection.

Weaknesses of our study are the lack of information on year of HIV seroconversion and on the presence of KS herpesvirus co-infection. Furthermore, we evaluated HAART use by intention-to-treat, that is, without subtracting all periods where treatment had been stopped, so its efficacy may be underestimated. A major strength of our cohort study is that it is the largest ever reported with respect to the number of KS cases and the number of person-years of HAART use. Furthermore, the representativeness of the SHCS with respect to Swiss PWHA was especially good (i.e., inclusion of 49% of all HIV-positive people and 67% of all AIDS cases in the country, www.shcs.ch).

## Figures and Tables

**Table 1:** Incidence rates and HR of KS overall and by selected characteristics, and use of HAART

	**HAART non-users^a^**				**HAART users**			
	**KS**	**py**	**Incidence/1000 py (95% CI)^b^**	**HR (95% CI)^c^**	**KS**	**py**	**Incidence/1000 py (95% CI)^b^**	**HR (95% CI)^c^**
Overall	545	37 861	15.0 (13.8–16.3)	1^d^	52	35 551	1.3 (1.0–1.7)	0.11 (0.08–0.14)
								
*HIV transmission category*								
MSM^d^	446	10 900	27.8 (25.3–30.5)	1	35	12 216	1.6 (1.1–2.3)	1
Het/Oth	62	10 183	8.9 (6.8–11.4)	0.27 (0.20–0.36)	16	13 796	1.2 (0.7–1.9)	0.54 (0.27–1.10)
IDU	37	16 778	2.1 (1.5–2.9)	0.09 (0.06–0.13)	1	9539	0.0 (0.0–0.3)	0.05 (0.01–0.37)
								
*Age at enrolment (years)*								
<35^d^	256	27 795	7.4 (6.5–8.4)	1	25	20 136	0.9 (0.6–1.3)	1
⩾35	289	10 066	17.6 (15.7–19.8)	1.53 (1.29–1.82)	27	15 416	1.4 (1.0–2.1)	1.07 (0.61–1.85)
								
*Place of birth*								
Europe^d^	529	36 334	15.0 (13.8–16.4)	1	43	32 495	1.0 (0.7–1.4)	1
Africa/Middle East	16	1527	12.5 (7.1–20.4)	1.84 (1.10–3.06)	9	3057	2.4 (1.1–4.6)	6.49 (2.79–15.11)
								
*CD4 cell count at enrolment (cells μl*^−*1*^)								
⩾350^d^	128	20 988	6.7 (5.6–8.0)	1	18	15 212	1.0 (0.6–1.6)	1
200–349	93	6126	15.3 (12.4–18.8)	2.44 (1.86–3.20)	9	8382	0.9 (0.4–1.8)	0.84 (0.38–1.88)
50–199	133	3883	33.6 (28.2–39.9)	5.04 (3.90–6.51)	11	7556	1.1 (0.6–2.0)	1.13 (0.53–2.44)
<50	94	1119	77.1 (62.3–94.3)	12.85 (9.59–17.23)	12	3021	4.8 (2.5–8.4)	3.26 (1.53–6.91)
Unknown	97	5745	18.1 (14.7–22.1)	—	2	1381	0.7 (0.1–2.7)	—
								
*Treatment interruption* e								
No^d^	—	—	—	—	28	27 234	0.9 (0.6–1.2)	1
Yes	—	—	—	—	24	8317	2.8 (1.8–4.1)	8.14 (4.01–16.54)

CI=confidence interval; HAART=highly active antiretroviral therapy; Het/Oth=heterosexuals and other; HR=hazard ratio; IDU=intravenous drug users; KS=Kaposi sarcoma; MSM=men having sex with men; py=person-years.

a Individuals who were never treated with HAART and py before HAART among HAART users.

b Rates are standardised (direct method) on age and/or gender based on all SHCS participants.

c Adjusted for centre, age, gender, and HIV transmission category, when appropriate.

d Reference category.

e Absence of any antiretroviral drug for ⩾3 months.
